# Ingested Nitrate and Breast Cancer in the Spanish Multicase-Control Study on Cancer (MCC-Spain)

**DOI:** 10.1289/ehp.1510334

**Published:** 2016-03-04

**Authors:** Nadia Espejo-Herrera, Esther Gracia-Lavedan, Marina Pollan, Nuria Aragonés, Elena Boldo, Beatriz Perez-Gomez, Jone M. Altzibar, Pilar Amiano, Ana Jiménez Zabala, Eva Ardanaz, Marcela Guevara, Antonio J. Molina, Juan Pablo Barrio, Ines Gómez-Acebo, Adonina Tardón, Rosana Peiró, Maria Dolores Chirlaque, Margarita Palau, Montse Muñoz, Laia Font-Ribera, Gemma Castaño-Vinyals, Manolis Kogevinas, Cristina M. Villanueva

**Affiliations:** 1 ISGlobal Centre for Research in Environmental Epidemiology (CREAL), Barcelona, Spain; 2 Departament de Ciències Experimentals i de la Salut, Universitat Pompeu Fabra, Barcelona, Spain; 3 CIBER Epidemiología y Salud Pública (CIBERESP), Madrid, Spain; 4 Cancer and Environmental Epidemiology Unit, National Centre for Epidemiology, Carlos III Institute of Health, Madrid, Spain; 5 Cancer Epidemiology Research Group, Oncology and Hematology Area, Instituto de Investigación Sanitaria (IIS) Puerta De Hierro, Madrid, Spain; 6 Public Health Division of Gipuzkoa, Biodonostia Research Institute, San Sebastian, Spain; 7 Navarra Public Health Institute, Pamplona, Spain; 8 Navarra Institute for Health Research (IdiSNA), Pamplona, Spain; 9 Research Group in Gene–Environment–Health Interactions (GIIGAS), University of Leon, León, Spain; 10 IDIVAL (Valdecilla Institute of Research), University of Cantabria, Santander, Spain; 11 Oncology Institute IUOPA (Institute of Oncology of Asturias), Universidad de Oviedo, Asturias, Spain; 12 Centre for Research in Public Health, Valencia, Spain; 13 Department of Epidemiology, Murcia Health Council, IMIB-Arrixaca, Murcia, Spain; 14 Department of Health and Social Sciences, Universidad de Murcia, Murcia, Spain; 15 Division of Public Health Quality and Innovation, Health Ministry of Spain, Madrid, Spain; 16 Translational Genomics and Targeted Therapeutics in Solid Tumors (IDIBAPS), Hospital Clinic, Barcelona, Spain; 17 IMIM (Hospital del Mar Medical Research Institute), Barcelona, Spain

## Abstract

**Background:**

Ingested nitrate leads to endogenous formation of N-nitroso compounds that are breast carcinogens in animals, but human evidence is limited.

**Objective:**

We evaluated ingested nitrate as a risk factor for breast cancer (BC) in a multicase–control study.

**Methods:**

Hospital-based incident BC cases and population-based controls were recruited in eight Spanish regions in 2008–2013; participants provided residential and water consumption from 18 years of age and information on known BC risk factors. Long-term nitrate levels (1940–2010) were estimated and linked with residential histories and water consumption to calculate waterborne ingested nitrate (milligrams/day). Dietary ingested nitrate (milligrams/day) was calculated using food frequency questionnaires and published dietary nitrate contents. Interactions with endogenous nitrosation factors and other variables were evaluated. A total of 1,245 cases and 1,520 controls were included in the statistical analysis.

**Results:**

Among the study regions, average ± SD waterborne ingested nitrate ranged from 2.9 ± 1.9 to 13.5 ± 7.5 mg/day, and dietary ingested nitrate ranged from 88.5 ± 48.7 to 154 ± 87.8 mg/day. Waterborne ingested nitrate was not associated with BC overall, but among postmenopausal women, those with both high nitrate (> 6 vs. < 2.6 mg/day) and high red meat intake (≥ 20 vs. < 20 g/day) were more likely to be cases than women with low nitrate and low red meat intake (adjusted odds ratio = 1.64; 95% confidence interval: 1.08, 2.49; overall interaction p-value = 0.17). No association was found with dietary nitrate.

**Conclusions:**

Waterborne ingested nitrate was associated with BC only among postmenopausal women with high red meat consumption. Dietary nitrate was not associated with BC regardless of the animal or vegetable source or of menopausal status.

**Citation:**

Espejo-Herrera N, Gracia-Lavedan E, Pollan M, Aragonés N, Boldo E, Perez-Gomez B, Altzibar JM, Amiano P, Zabala AJ, Ardanaz E, Guevara M, Molina AJ, Barrio JP, Gómez-Acebo I, Tardón A, Peiró R, Chirlaque MD, Palau M, Muñoz M, Font-Ribera L, Castaño-Vinyals G, Kogevinas M, Villanueva CM. 2016. Ingested nitrate and breast cancer in the Spanish Multicase-Control Study on Cancer (MCC-Spain). Environ Health Perspect 124:1042–1049; http://dx.doi.org/10.1289/ehp.1510334

## Introduction

Breast cancer (BC) is the leading cause of cancer mortality and is the most common cancer among women worldwide. In Spain, 25,215 new cases are diagnosed annually (Ferlay et al. 2013), and incidence rates have increased over the last three decades (Pollán et al. 2009). Several risk factors for BC have been identified, including sex, age, nulliparity, short breastfeeding, menstrual and reproductive history, high body mass index (particularly in postmenopausal women), physical inactivity, high alcohol or energy intake, use of drugs with estrogenic action, exposure to ionizing radiation, specific genetic factors, family history of BC, previous diagnosis of non-malignant breast diseases, and high mammographic density (Hankinson et al. 2004; Romieu et al. 2015; Stewart and Wild 2014). Established risk factors explain ~50% of the incidence variation of this malignancy, and other environmental exposures may partly explain the remaining variation (Brody et al. 2007).

Nitrate is a frequent contaminant in drinking water worldwide; its presence is related to excessive fertilizer use or to sewage (Wakida and Lerner 2005). Humans are exposed to nitrate through diet and through drinking water ingestion. The maximum nitrate level in drinking water [50 mg/L as nitrate ion (NO_3_^–^) or 10 mg/L of nitrate-nitrogen (nitrate-N)] (EU 1998; WHO 2008b) was established to prevent acute health effects in children (methemoglobinemia), but the effects of long-term exposure to lower levels, including cancer risk, are not well established (Ward et al. 2005).

Ingested nitrate is classified as a probable human carcinogen in conditions of endogenous nitrosation (IARC 2010). This process involves the conversion of nitrate into nitrite and the synthesis of *N*-nitroso compounds (NOCs) in the gastrointestinal tract. The intake of antioxidant vitamins and the use of nonsteroidal antiinflammatory drugs (NSAIDs) inhibit endogenous nitrosation, whereas meat intake and inflammatory gastrointestinal conditions promote it (Ward et al. 2005). NOCs are potent carcinogens for several animal species (Lijinsky et al. 1992). Some NOCs, such as *N*-methyl-*N*-nitrosourea (MNU), are used to induce BC in experimental animal studies, and young rats exposed to MNU were more susceptible to developing breast tumors (Tsubura et al. 2011). In cell-based studies, low doses of nitrite and nitrate were able to mimic estradiol and to activate estrogen receptors, suggesting a potential role of these anions in the etiology or progression of cancer (Veselik et al. 2008).

Despite the evidence in animals, few epidemiologic studies have evaluated the association between exposure to nitrate or to its derivatives and BC. Relevant studies were conducted in the United States (Brody et al. 2006; Weyer et al. 2001); these studies did not find associations between waterborne or dietary ingested nitrate and BC. A recent cohort study of postmenopausal women in the United States reported that BC was increased in the highest versus lowest quintile of water nitrate intake among women who also had folate ingestion of ≥ 400 μg/day, but the study did not find any association with dietary nitrate (Inoue-Choi et al. 2012). The authors of previous studies attributed their null associations to limitations in the exposure assessment (i.e., a lack of data on water daily intake), to the coexistence of antioxidants (i.e. vitamin C) in main dietary sources of nitrate (vegetables), and to the lack of evaluation of nitrate intake from specific dietary sources such as animal foods and processed meat. In summary, human evidence relating nitrate exposure and BC is limited and inconclusive. Studies evaluating different exposure windows, including individual water consumption information, endogenous nitrosation factors and other covariables, are required to enhance the available evidence.

In the present study, we aimed to evaluate nitrate ingested through drinking water and diet as a risk factor for BC in a population-based multicase–control study conducted in Spain (MCC-Spain).

## Methods

### Study Design and Population

This study is part of the MCC-Spain study, which aims to evaluate the influence of environmental exposures on common cancers in Spain (e.g., female breast or colorectal). The study population was recruited between 2008 and 2013 in eight Spanish provinces (see Table 1). Cases were identified shortly after diagnosis (average: 3.2 months, SD 4.2 months) through an active search by periodic visits to the collaborating hospital departments (i.e., gynecology, oncology, general surgery, radiotherapy, and pathology departments). Participant hospitals were the reference centers for oncologic diseases in each study area. Only incident cases who were diagnosed within the recruitment period, without malignant BC history, between 20 and 85 years of age, resided in the hospitals’ catchment areas for at least 6 months prior to recruitment, and were able to answer the epidemiological questionnaire (Castaño-Vinyals et al. 2015) were included. All cases had histological confirmation and included all malignant BC [*International Classification of Diseases* (10th Revision) (World Health Organization 2008a); ICD-10: C50] and frequent *in situ* breast cancers (ICD-10:D05.1, D05.7). Population-based controls were frequency-matched to cases by age, sex, and region, ensuring at least one control of the same sex and 5-year interval age for each case. Eligible controls were randomly selected from administrative records of primary care health centers located within hospitals’ catchment areas. For each control needed, five potential participants of similar age, sex, and hospital catchment area were randomly selected from the lists of general practitioners. If contact with the first person on the list was not achieved (after at least five attempts made at different times of the day), or if he/she refused to participate, the next person on the list was approached. The study protocol was approved by the ethics review board from each participating center, and participants signed an informed consent before recruitment.

**Table 1:** Characteristics of the study population (1,245 cases*^a^* and 1,520 controls).

Characteristic	Cases *n* (%)	Controls *n* (%)	*p*-Value (χ^2^ test)
Study area
Asturias	62 (5.0)	107 (7.0)
Barcelona	256 (20.6)	342 (22.5)
Cantabria	103 (8.3)	134 (8.8)
Gipuzkoa	171 (13.7)	246 (16.2)
León	155 (12.4)	168 (11.0)
Madrid	274 (22.0)	311 (20.5)
Navarra	171 (13.7)	158 (10.4)
Valencia	53 (4.3)	54 (3.6)
Age (years)
Mean (SD)	56.7 (12.3)	59.8 (12.9)
Range	23–85	24–85
≤ 50	430 (34.5)	417 (27.4)
51–60	352 (28.3)	354 (23.3)
61–70	284 (22.8)	379 (24.9)
> 70	179 (14.4)	370 (24.3)
Education
< Primary school	184 (14.8)	266 (17.5)
Primary school	416 (33.4)	483 (31.8)
Secondary school	410 (32.9)	461 (30.3)
University	235 (18.9)	310 (20.4)
Body mass index (kg/m^2^)	
< 18.5	22 (1.8)	32 (2.1)
18.5–24.9	555 (44.6)	717 (47.2)
25–29.9	442 (35.5)	498 (32.8)
≥ 30	226 (18.2)	273 (18.0)	0.40
Family history of BC^*a*^	
No	816 (67.5)	1,189 (81.4)
Yes	393 (32.5)	271 (18.6)	< 0.001
Missing	36	60
Menopausal status
Premenopausal	332 (26.7)	347 (22.8)
Postmenopausal	913 (73.3)	1,173 (77.2)	0.02
Age at menopause^*b*^	
Mean (SD) (years)	48.9 (5.2)	48.6 (5.2)
Range (years)	28–59	22–62
≤ 50 years old	460 (57.9)	634 (64.4)
> 50 years old	335 (42.1)	351 (35.6)	0.01
Missing	450	535
Age at menarche (years)	
Mean (SD)	12.8 (1.6)	12.9 (1.6)
Range	8–20	7–20
≤ 12	535 (43.5)	608 (42.0)
13–14	562 (45.7)	661 (45.7)
≥ 15	133 (10.8)	178 (12.3)	0.45
Missing	15	73
Age at first birth^*c*^ (years)	
Mean (SD)	27 (4.9)	26.8 (4.6)
Range	16–42	15–43
≤ 30	763 (77.7)	1,005 (80.7)
> 30	219 (22.3)	241 (19.3)	0.09
Missing	263	274
Nulliparity
No	989 (79.6)	1,251 (82.6)
Yes	253 (20.4)	264 (17.4)	0.05
Missing	3	5
Oral contraceptive use	
Never	663 (53.4)	787 (51.8)
Ever	579 (46.6)	732 (48.2)	0.41
Missing	3	1
Smoking
No	670 (54.1)	905 (59.6)
Yes	569 (45.9)	613 (40.4)	0.003
Missing	6	2
Alcohol intake^*d*^
No	384 (35.4)	516 (38.5)
Yes	700 (64.6)	824 (61.5)	0.20
Missing	161	180
Energy intake (kcal/day)	
≤ 1,479	288 (26.6)	447 (33.4)
> 1,479–1,894	362 (33.4)	447 (33.3)
> 1,894	434 (40.0)	446 (33.3)	< 0.001
Missing	161	180
Vitamin C intake (mg/day)	
≤ 129	396 (36.5)	447 (33.4)
> 129–203	317 (29.2)	447 (33.3)	
> 203	371 (34.2)	446 (33.3)	0.08
Missing	161	180
Vitamin E intake (mg/day)	
≤ 8.6	356 (32.8)	447 (33.4)
> 8.6–12.2	354 (32.7)	447 (33.3)
> 12.2	374 (34.5)	446 (33.3)	0.82
Missing	161	180
Folate intake (μg/L)	
≤ 252	335 (30.9)	447 (33.4)
> 252–340	370 (34.1)	447 (33.3)
> 340	379 (35.0)	446 (33.3)	0.42
Missing	161	180
Vegetable intake^*e*^**(g/day)	
≤ 422	393 (36.3)	447 (33.4)
> 422–642	340 (31.4)	447 (33.3)
> 642	351 (32.4)	446 (33.3)	0.31
Missing	161	180
Red meat intake (g/day)	
≤ 16	311 (28.7)	447 (33.4)
> 16–29	346 (31.9)	447 (33.3)
> 29	427 (39.4)	446 (33.3)	0.01
Missing	161	180
Processed meat intake (g/day)			
≤ 5.2	293 (27.0)	447 (33.4)
> 5.2–13.4	360 (33.2)	447 (33.3)
> 13.4	431 (39.8)	446 (33.3)	0.001
Missing	161	180
Interview quality
Unsatisfactory	3 (0.2)	1 (0.1)
Questionable	92 (7.5)	87 (6.1)
Reliable	562 (46.0)	736 (51.8)
High quality	564 (46.2)	596 (42.0)	0.02
Missing	24	100
Histological type
Ductal	951 (76.4)
Others	162 (13.0)
Undefined	132 (10.6)
Estrogen receptors
Positive	990 (79.5)
Negative	218 (17.5)
Undefined	37 (2.9)
^***a***^Breast cancer cases. ^***b***^Distribution only among postmenopausal women. ^***c***^Distribution among parous women. ^***d***^Alcohol intake from 30 to 40 years of age. ^***e***^Vegetable intake includes vegetables and fruits.			

### Questionnaires and Response Rates

A structured computerized questionnaire was administered by trained personnel in face-to-face interviews (http://www.mccspain.org). Collected data included *a*) sociodemographic characteristics; *b*) lifetime residential history; *c*) type of water consumed in each residence (municipal/bottled/well/other); *d*) amount of water intake at home, including water *per se*, coffee, tea, and other water-based beverages; *e*) smoking habits; *f*) history of gastric ulcers and use of nonsteroidal antiinflammatory drugs (NSAIDs); *g*) gynecologic and reproductive history; *h*) use of oral contraceptives (OCs) or hormonal replacement therapy (HRT); and *i*) physical activity. Anthropometric measurements were self-reported (weight, height) or measured (waist and hip circumference) during the interview. Histological type and estrogen receptor data were available for cases. Average response rates differed among regions and were 71% among cases and 53% among controls overall (Castaño-Vinyals et al. 2015). In total, 1,585 cases and 1,822 controls were recruited and answered the questionnaire.

Dietary information corresponding to 1 year before recruitment for controls or to 1 year before diagnosis for cases, was collected using a validated food frequency questionnaire (FFQ) (Martin-Moreno et al. 1993). The FFQ comprised 140 food items, including regional Spanish products, and was either administered during the interview or self-administered and returned by mail. Instructions to complete the FFQ were provided during the interview. The FFQ was used to estimate the average daily intake of vegetables, fruits, meat, dairy products, and alcoholic beverages.

### Dietary Nitrate and Nutrient Estimates

Published food-composition tables (Farran et al. 2004) were used to calculate the daily intake of energy and nutrients (vitamins C, D, and E and folate). Dietary nitrate intake (milligrams/day) was estimated based on the average intake of food items (grams/day) and the published nitrate content (milligrams/100 grams) in food items including vegetables [European Food Safety Authority (EFSA) 2008], animal products, and others (Griesenbeck et al. 2009; Jakszyn et al. 2004). Nitrate contents were assigned to the following food items: 21 vegetables (including tubers), 13 fruits, 17 animal sources (including red, white, and processed meat and dairy products), frequently consumed foodstuffs (bread, rice, and pasta), and 1 alcoholic beverage (beer). For the calculations, “red meat” included beef, lamb, and pork meat; “processed meat” included bacon, hot dogs, smoked ham, Spanish cured ham, and other cured sausages.

### Nitrate Levels in Drinking Water

We collected environmental data from municipalities covering ≥ 80% of person-years in each area. We sent a standardized questionnaire to local authorities and water companies to ascertain current and historical nitrate measurements in water from municipal distribution systems and water source characteristics (surface/groundwater proportion). Monitoring levels (2004–2010) were provided by the Sistema de Información Nacional en Aguas de Consumo (SINAC). Measurements below the quantification limits (QL) (5% of measurements) were imputed as half of the QL value. If the QL was missing, the value was imputed as half of the value of the most frequently reported QL (1.0 mg/L).

We measured nitrate levels in samples of the most-consumed bottled water brands in Spain (Espejo-Herrera et al. 2013). Nitrate levels in wells and springs not covered by the municipal water distribution system were measured in September 2013 (unpublished data). A total of 28 water samples were collected in 21 municipalities of the León region, where nonmunicipal water consumption was the highest among our study areas (26% of controls in the longest residence).

### Estimation of Long-Term Nitrate Levels in Drinking Water

We calculated annual average nitrate levels back to 1940 by water zone (defined as a geographical area supplied by water with a homogeneous source and quality) that usually corresponded to municipality. We calculated annual averages based on available nitrate measurements. For years without measurements, we assigned the average of the total measurements available in the water zone, as long as the water source remained constant. In cases where the water source changed, the ground water percentage was used as a weight to modulate the estimations, assuming that nitrate levels were higher at higher ground water proportions. In municipalities without any nitrate measurement (covering 0.5% of the total person-years), we imputed the levels of neighboring municipalities supplied with similar ground water proportion ± 10%.

### Individual Exposure Variables

We linked nitrate levels in drinking water (measured and imputed) and residential histories by year and municipality covering the exposure window from 18 years of age to 2 years before the study interview (henceforth referred to as “adult life” or “long-term exposure”). To calculate waterborne ingested nitrate (milligrams/day), we assigned nitrate levels [milligrams/liter nitrate ion (NO_3_^–^)] in drinking water by year according to the water type consumed. Nitrate levels in municipal water (residential levels) were assigned for tap-water consumption. Levels in the sampled bottled waters were averaged using the sales frequency of each brand as a weight. This weighted average (6.1 mg/L of NO_3_^–^) was assigned when bottled water consumption was reported. Levels in well-water samples from León (range: 0.5–93 mg/L) were assigned to women reporting well-water consumption in this area according to the postal code of the residence. Nitrate levels in well water were not available for other areas, and waterborne ingested nitrate was considered missing for years when well-water consumption was reported among women from those areas (range: 0.6–8.2% of controls in the longest residence). The annual nitrate estimates were averaged and multiplied by the average daily water intake at home (1.3 ± 0.7 L/day in cases, 1.2 ± 0.7 L/day in controls). Water intakes > 99th percentile (4 L/day), considered implausible, were treated as missing values in the analyses. We also calculated the average waterborne ingested nitrate in two alternative exposure periods: from 15 to 2 years before the interview (“recent” exposure), and from 18 to 30 years of age (“early adulthood” exposure).

In a subset of participants from Barcelona with information on water type changes within residences, 86% of subjects reporting bottled water consumption in the last residence actually switched from municipal to bottled water after the year 2000. Potential misclassification of the water type consumed (municipal/bottled), particularly in recent residences, was a concern. To address this issue, we calculated an alternative variable for waterborne ingested nitrate in adult life. We assumed that women reporting bottled water consumption and living during at least 10 years in the last residence (or in the previous one), actually consumed municipal water before the year 2000 and bottled water thereafter.

### Statistical Analyses

The population analyzed (1,245 cases, 1,520 controls) included women with data on both waterborne ingested nitrate covering ≥ 70% of the main exposure period (from 18 years of age to 2 years before the interview) and on daily water intake. We estimated odds ratios (ORs) and 95% confidence intervals (CIs) of BC for categorized nitrate intake using unconditional logistic regression. Categories of exposure (quartiles or tertiles) were specifically defined for pre- and postmenopausal women according to the distribution in controls. Basic models were adjusted for age (continuous), study area, and education (three categories: ≤ primary, high school, and ≥ university). Several potential confounders were explored separately for pre- and postmenopausal women, including smoking (yes/no 5 years before recruitment), average leisure physical activity from 16 years of age until 2 years before the interview [measured in metabolic equivalents of task (METS)/hour/week], body mass index (BMI), family history of malignant BC in any blood relative (yes/no), NSAID use (yes/no), age at menarche, age at menopause (both, continuous variables in years and categorized variables). Menopause and age at menopause were defined according to the date of the last regular menstrual period. Age at first birth, nulliparity (yes/no), parity (number of births), total months of breastfeeding (categorized), OC and HRT use (never/ever), intake of alcohol (no/yes at 30 years of age), intake of energy and folate (tertiles), and endogenous nitrosation modulators (intake of vitamin C, vitamin E, red meat, and processed meat) were also explored as potential confounders. Only established BC risk factors (Stewart and Wild 2014), and variables that changed the risk estimates > 10% were included in the adjustment (age, study area, education, BMI, family history of BC, age at first birth, use of OC, energy intake and age at menopause for postmenopausal women). For each model covariate, missing data in categorical variables were classified as a separate category in multivariate analyses. Trend *p*-values were derived from a likelihood ratio test (LRT) comparing a model having the categorical nitrate variable as an ordinal variable (0, 1, 2) with a model that excluded the variable.

We used generalized additive models (GAMs) to evaluate the exposure–response relationship between waterborne nitrate intake and BC by study area. We stratified analyses for waterborne ingested nitrate by relevant covariates, including endogenous nitrosation factors (intake of vitamin C, vitamin E, red meat, and processed meat), folate intake, and smoking. Strata of continuous variables were defined according to the distribution in controls (≤ or > median). We compared the multivariate models with and without the interaction term using an LRT, and *p* values < 0.10 were considered indicative of multiplicative interaction. Analyses by histological type [ductal ICD-10: C50 and other *in situ* tumors (ICD-10: D05.1, D05.7)] and by estrogen receptor (ER) status were also conducted. In sensitivity analyses, we used the alternative variables of waterborne ingested nitrate. We also excluded women with missing data in covariables and women with unsatisfactory or missing interview quality. Interview quality was assessed by the interviewers as unsatisfactory, questionable, reliable, or high quality based on the completeness of the information provided. All statistical analyses were performed using STATA version 12.0 (StataCorp LP).

## Results

General characteristics of the study population are shown in Table 1. Compared with controls, cases showed higher frequency of family history of BC; age at menopause > 50 years; age at first birth > 30 years; higher intake of energy, red meat, and processed meat; lower intake of vitamin C; and nulliparity (*p*-value < 0.05 in *χ* ^2^ test). Among the women analyzed, 24.6% (*n* = 679) were premenopausal and 75.4% (*n* = 2,086) were postmenopausal. Women with assigned nitrate levels in drinking water for < 70% of their residential history in adult life and those without information on daily water intake were excluded from the analyses. Compared with those who were excluded, the women who were analyzed showed a higher proportion of controls (55% vs. 47%) and postmenopausal women (75.4% vs. 63.9%); a lower proportion of university education and nulliparity; were older; and had a lower intake of vitamins C and E; however, their levels of waterborne ingested nitrate were similar (see Table S1).

**Figure 1:**
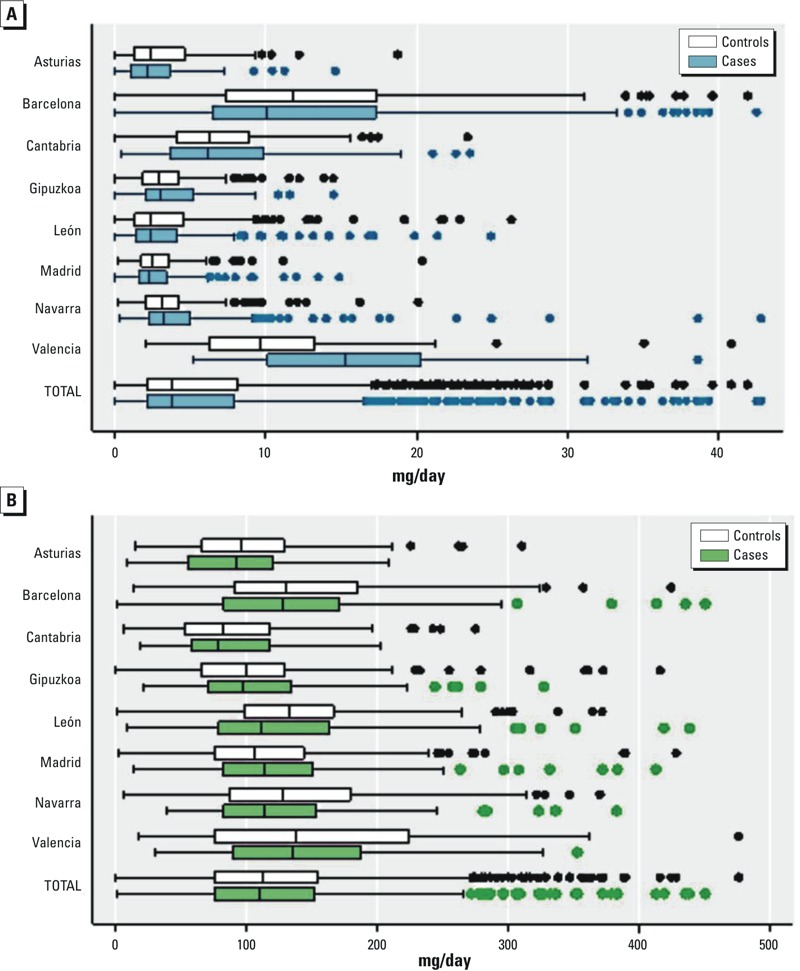
Ingested nitrate levels (milligrams/day) through drinking water from 18 years of age to 2 years before the interview (*A*) and diet (*B*) across study areas. Women with waterborne ingested levels > 44 mg/day (*n *= 6) or with dietary ingested levels > 476 mg/day (*n *= 7) were excluded from the graphics. Boxes extend from the 25th to the 75th percentile. Horizontal bars represent the median, whiskers indicate the 10th and 90th percentiles, and outliers are represented as points.

Figure 1 shows the average ingested nitrate levels in adult life for cases and controls. Across the investigated areas, levels of waterborne ingested nitrate (mean ± SD) ranged from 2.9 ± 1.9 to 13.5 ± 7.5 mg/day (Figure 1A) and were higher among post versus premenopausal women (6.74 ± 7.1 vs. 5.12 ± 5.6 mg/day; *p-*value < 0.001 for Mann–Whitney U test). Ingested levels during alternative exposure periods (from 15 to 2 years before the study interview and from 18 to 30 years of age) were similar to the levels presented in Figure 1A (results not shown). Across the investigated areas, dietary ingested nitrate levels (mean ± SD) ranged from 88.5 ± 48.7 to 154 ± 87.8 mg/day (Figure 1B) and were higher among post- versus premenopausal women (129.0 ± 86.2 vs. 109.7 ± 62.1 mg/day; *p*-value < 0.001 for *t*-test). On average, 6.0% ± 7.0 of the total dietary nitrate was derived from animal sources, 84.7% ± 12.1 from vegetables, and the remaining portion from other food products (e.g., grains). Ingested nitrate from animal sources (mean ± SD: 5.5 ± 2.9 mg/day) was greater among pre- versus postmenopausal women (5.9 ± 2.7 vs. 5.2 ± 3.0 mg/day; *p-*value < 0.0001 for *t-*test), but ingested nitrate from vegetable sources (mean ± SD: 110 ± 79.6 mg/day) was lower among pre- versus postmenopausal women (96.5 ± 60.4 vs. 115.0 ± 84.5 mg/day; *p-*value < 0.0001 for *t-*test) (results not shown).

Table 2 shows the association between waterborne ingested nitrate and BC. Among postmenopausal women, the fully adjusted OR (95% CI:) was 1.29 (0.92, 1.81) for > 8.8 mg/day compared with the lowest intake levels (< 2.3 mg/day). After excluding postmenopausal women with missing or unreliable interview quality (*n* = 118), the OR (95% CI:) was 1.32 (0.93, 1.86) for > 8.8 versus < 2.3 mg/day. Among premenopausal women, the fully adjusted OR (95% CI:) was 1.14 (0.67, 1.94) for > 6.3 mg/day compared with the lowest intake levels (< 1.8 mg/day), and the results were similar after excluding premenopausal women with unreliable interviews (*n* = 10). The results were also similar when waterborne exposure from 18 years of age to 2 years before the study interview was defined assuming bottled water use after 2000, for exposures from 15 to 2 years before the study interview, and from 18–30 years of age (see Table S2). Exposure–response curves among study areas did not show associations except at the highest levels, where estimates were extremely imprecise (see Figure S1).

**Table 2:** Waterborne ingested nitrate from 18 years of age to 2 years before study interview and breast cancer associations by menopausal status. Odds ratios (ORs) and 95% confidence intervals (CIs).

Menopausal status	Cases (*n*)	Controls (*n*)	OR^*a*^ (95% CI)	OR^*b*^ (95% CI)	Cases^*c*^ (*n*)	Controls^*c*^ (*n*)	OR^*b*^ (95% CI)
Postmenopausal	913	1,173			888	1,080
< 2.3 mg/day	227	294	Reference	Reference	222	289	Reference
≥ 2.3–4.0 mg/day	232	293	1.06 (0.82, 1.36)	1.09 (0.84, 1.41)	229	285	1.11 (0.86, 1.45)
> 4.0–8.8 mg/day	222	293	1.08 (0.82, 1.41)	1.07 (0.81, 1.42)	213	263	1.10 (0.83, 1.46)
> 8.8 mg/day	232	293	1.28 (0.92, 1.77)	1.29 (0.92, 1.81)	224	243	1.32 (0.93, 1.86)
*p *for trend			0.19	0.20			0.16
Premenopausal	332	347			330	339
< 1.8 mg/day	72	87	Reference	Reference^*d*^	72	86	Reference^*d*^
≥ 1.8–3.1 mg/day	87	87	1.24 (0.79, 1.92)	1.31 (0.83, 2.06)	86	86	1.28 (0.81, 2.02)
> 3.1–6.3 mg/day	85	87	1.08 (0.68, 1.72)	1.03 (0.64, 1.66)	85	85	0.99 (0.61, 1.60)
> 6.3 mg/day	88	86	1.11 (0.66, 1.86)	1.14 (0.67, 1.94)	87	82	1.05 (0.61, 1.80)
*p *for trend			0.78	0.80			0.97
Pre + Postmenopausal	1,245	1,520			1,218	1,419
< 2.2 mg/day	319	380	Reference	Reference	315	374	Reference
≥ 2.2–3.8 mg/day	303	380	0.96 (0.77, 1.19)	0.98 (0.79, 1.23)	298	373	0.98 (0.78, 1.22)
> 3.8–8.1 mg/day	316	380	1.04 (0.83, 1.31)	1.01 (0.80, 1.28)	308	351	1.01 (0.80, 1.28)
> 8.1 mg/day	307	380	1.09 (0.83, 1.43)	1.08 (0.82, 1.43)	297	321	1.08 (0.81, 1.44)
*p* for trend			0.53	0.64			0.63
Trend *p*-values were derived from a likelihood ratio test that compared a model including the categorical nitrate intake variable as an ordinal variable (0, 1, 2) with a model that excluded this variable. ^***a***^Adjusted for study area, age, and education. ^***b***^Adjusted for study area, age, education, body mass index, family history of breast cancer, age at first birth, age at menopause, use of oral contraceptives, and energy intake. ^***c***^Women with unreliable interviews or missing data on interview quality (postmenopausal, *n *= 118; premenopausal, *n *= 10) were excluded. ^***d***^Age at menopause was excluded from the adjustment for premenopausal women.							

Table 3 shows the associations between waterborne ingested nitrate and BC for postmenopausal women across categories of relevant covariables. BC was inversely associated with high vitamin C + E intake (> 181 mg/day) versus low vitamin C + E intake among women with low waterborne nitrate intake (< 2.6 mg/day) (OR = 0.60; 95% CI: 0.39, 0.92), and the overall interaction *p-*value was 0.08. However, there was no evidence that vitamin C + E intake modified the odds of BC among those in the second or third tertile of waterborne nitrate. This inverse association was not observed when vitamins C and E were analyzed separately. BC was more common among women with the highest waterborne nitrate (> 6 mg/day) and the highest red meat intake (> 20 g/day) than among women with low waterborne nitrate (< 2.6 mg/day) and low red meat intake (OR = 1.64; 95% CI: 1.08, 2.49). BC was not increased in women with high waterborne nitrate and low red meat intake (OR = 1.08; 95% CI: 0.72, 1.47), but the overall interaction between nitrate and red meat intake was not significant (LRT *p-*value = 0.17). The results for processed meat intake followed a similar pattern. BC was also more common among women with the highest waterborne nitrate intake and smoking history (OR = 1.48; 95% CI: 0.99, 2.21) than among women with low waterborne nitrate without smoking history, but the overall interaction was not significant (LRT *p-*value = 0.12).

**Table 3:** Interaction of waterborne ingested nitrate from age 18 to 2 years before study interview with relevant dietary covariables and breast cancer associations among postmenopausal women*^a^*.

Waterborne ingested nitrate	Cases (*n*)	Controls (*n*)	OR (95% CI)	Cases (*n*)	Controls (*n*)	OR^*b*^ (95% CI)	Interaction *p*-value^*c*^
Vitamin C			< 170 mg/day			≥ 170 mg/day
< 2.6 mg/day	140	162	Reference	104	161	0.73 (0.51, 1.05)
≥ 2.6–6.0 mg/day	118	169	0.88 (0.62, 1.24)	153	171	1.08 (0.77, 1.52)
> 6.0 mg/day	146	185	1.19 (0.80, 1.77)	136	183	1.06 (0.72, 1.58)	0.10
Vitamin E			< 10 mg/day			≥ 10 mg/day
< 2.6 mg/day	131	170	Reference	113	153	0.84 (0.58, 1.22)
≥ 2.6–6.0 mg/day	132	160	1.18 (0.83, 1.67)	139	180	0.94 (0.65, 1.34)
> 6.0 mg/day	123	186	1.16 (0.77, 1.74)	159	182	1.25 (0.82, 1.88)	0.41
Vitamin C + E			< 181 mg/day			≥ 181 mg/day
< 2.6 mg/day	142	161	Reference	102	162	0.60 (0.39, 0.92)
≥ 2.6–6.0 mg/day	116	167	0.94 (0.62, 1.42)	155	173	1.09 (0.73, 1.63)
> 6.0 mg/day	145	185	1.17 (0.73, 1.89)	137	183	1.02 (0.64, 1.64)	0.08
Folate			< 300 μg/day			≥ 300 μg/day
< 2.6 mg/day	143	169	Reference	101	154	0.74 (0.52, 1.07)
≥ 2.6–6.0 mg/day	116	173	0.89 (0.63, 1.25)	155	167	1.09 (0.78, 1.54)
> 6.0 mg/day	135	174	1.19 (0.80, 1.77)	147	194	1.09 (0.73, 1.62)	0.13
Red meat			< 20 g/day			≥ 20 g/day
< 2.6 mg/day	104	151	Reference	140	172	1.03 (0.72, 1.47)
≥ 2.6–6.0 mg/day	119	160	1.13 (0.78, 1.63)	152	180	1.17 (0.82, 1.67)
> 6.0 mg/day	124	205	1.08 (0.71, 1.62)	158	163	1.64 (1.08, 2.49)	0.17
Processed meat			< 7.2 g/day			≥ 7.2 g/day
< 2.6 mg/day	118	180		126	143	1.09 (0.76, 1.56)
≥ 2.6–6.0 mg/day	116	165	1.12 (0.79, 1.60)	153	175	1.24 (0.88, 1.75)
> 6.0 mg/day	100	171	1.14 (0.76, 1.70)	182	197	1.62 (1.08, 2.44)	0.46
Smoking			No			Yes
< 2.6 mg/day	161	226	Reference	118	141	Reference
≥ 2.6–6.0 mg/day	188	247	1.12 (0.84, 1.51)	116	147	0.93 (0.66, 1.32)
> 6.0 mg/day	197	296	1.13 (0.80, 1.60)	128	115	1.48 (0.99, 2.21)	0.12
^***a***^Only women with complete information on dietary covariables (*n *= 1,828) or smoking (*n *= 2,080) were analyzed. ^***b***^Adjusted for study area, age, education, body mass index, family history of breast cancer, age at menopause, age at first birth, oral contraceptives use, and energy intake. ^***c***^*p*-Value for overall interaction was calculated by comparing the multivariate models with and without the interaction term using a likelihood ratio test.							

Stratified analyses among premenopausal women resulted in less-precise estimates of associations owing to smaller numbers of observations. Most of the ORs observed across strata were close to 1, and overall interactions were not significant (LRT *p*-values > 0.10) (see Table S3).

Among all BC cases, 951 (76.4%) were ductal (ICD-10 C50), 162 (13.0%) were other malignant and *in situ* cancers (ICD-10 D05.1, D05.7), and 132 (10.6%) were undefined. Regarding ER status, 990 (79.5%) were positive, 218 (17.5%) were negative, and 37 (2.9%) had missing ER status. Stratified analyses among pre- and postmenopausal women combined showed similar ORs for ductal and other/undefined tumors as well as for ER-positive and ER-negative cancers (see Table S4).

Overall, BC was not associated with dietary nitrate from animal or vegetable sources (Table 4). The ORs reported were similar after adjusting for endogenous nitrosation factors (intake of vitamin C, vitamin E, and red and processed meat) and other covariables listed in Table 1 (data not shown) and after excluding women with low interview quality (*n* = 128 among pre- and postmenopausal women). Similar results were observed in separate analyses for pre- and postmenopausal women (see Table S5).

**Table 4:** Odds ratio (OR) of breast cancer associated with dietary ingested nitrate (mg/day) from different sources (*n *= 2,424)*^a^*.

Ingested nitrate from	Cases (*n*)	Controls (*n*)	OR^*b*^ (95% CI)	OR^*c*^ (95% CI)
Animal sources
< 4.0 mg/day	307	447	Reference	Reference
4.0–< 6.0 mg/day	364	447	1.12 (0.92, 1.38)	1.02 (0.82, 1.26)
≥ 6.0 mg/day	413	446	1.19 (0.97, 1.47)	1.04 (0.83, 1.31)
*p *for trend			0.09	0.72
Vegetables sources
< 76 mg/day	385	447	Reference	Reference
76–122 mg/day	355	447	0.92 (0.75, 1.12)	0.90 (0.74, 1.11)
> 122 mg/day	344	446	0.90 (0.74, 1.11)	0.86 (0.69, 1.06)
*p *for trend			0.33	0.15
Total diet
< 90 mg/day	387	447	Reference	Reference
90–138 mg/day	349	447	0.90 (0.74, 1.10)	0.86 (0.70, 1.06)
> 138 mg/day	348	446	0.90 (0.73, 1.10)	0.83 (0.67, 1.03)
*p *for trend			0.30	0.09
Total diet + waterborne
< 96 mg/day	386	447	Reference	Reference
96–144 mg/day	341	447	0.89 (0.73, 1.09)	0.84 (0.69, 1.04)
> 144 mg/day	357	446	0.94 (0.76, 1.15)	0.87 (0.70, 1.08)
*p *for trend			0.51	0.19
Trend *p*-values derived from a likelihood ratio test that compared a model including the categorical nitrate intake variable as an ordinal variable (0, 1, 2) with a model that excluded this variable. ^***a***^Only women with available data from the food frequency questionnaire were analyzed. ^***b***^Adjusted for study area, age, and education. ^***c***^Adjusted for study area, age, education, body mass index, family history of breast cancer, use of oral contraceptives, and energy intake.				

## Discussion

Average waterborne ingested nitrate levels from 18 years of age to 2 years before the interview was 6.2 ± 6.2 mg/day among controls and 6.6 ± 7.4 mg/day among cases. These levels were not associated with BC overall. However, in postmenopausal women, BC was significantly increased (*p* < 0.05) in women in the highest tertile of waterborne nitrate and with high red meat intake compared with women in the lowest tertile of waterborne nitrate and with low red meat intake. Dietary ingested nitrate (mean ± SD: 125.7 ± 80.3 mg/day in controls and 123.2 ± 82.3 mg/day in cases) was not associated with BC among pre- or postmenopausal women regardless of the vegetable or animal source.

To our knowledge, this is the first case–control study on ingested nitrate and BC in a European population. Most previous studies of waterborne nitrate exposure and BC have reported null associations (Brody et al. 2006; Weyer et al. 2001). A recent cohort study conducted in postmenopausal women from the state of Iowa (*n* = 2,875 BC cases in total), suggested an association between BC and waterborne nitrate intake in interaction with folate intake (Inoue-Choi et al. 2012). Individual data on daily water intake were not available in that study, but estimated waterborne nitrate intake levels were higher than the levels in our study (median: 20 mg/day vs. 3.8 mg/day, respectively), as was the folate intake (median: 350 μg/day vs. 300 μg/day, respectively). We did not confirm an interaction with folate, most likely because of the differences in nitrate and folate intake levels, as well as other differences including the cancer subtypes evaluated.

Analyses stratified by endogenous nitrosation factors (intake of vitamin C, vitamin E, and red and processed meat) and by other variables (listed in Table 1) did not show significant differences across categories; the CIs were overlapped and included the null value. BC occurred more frequently among postmenopausal women with the highest waterborne nitrate and red meat intake than among postmenopausal women with low waterborne nitrate intake and low red meat intake, and the overall interaction *p-*value was > 0.10. However, this joint effect is plausible because red meat contains amines, amides, and heme iron that may increase endogenous formation of NOCs (Bingham et al. 2002). The combined intake of vitamins C and E seemed to exert a protective effect that was limited to postmenopausal women in the lowest tertile of waterborne nitrate intake. These findings require confirmation in future studies because multiple stratifications were conducted, and chance cannot be ruled out.

The associations between waterborne ingested nitrate and BC were slightly higher in postmenopausal women than in premenopausal women. However, insufficient statistical power owing to small sample size may partly explain the null results among premenopausal women. We did not find an interaction between menopausal status and nitrate intake (*p*-value = 0.63) (data not shown), but we evaluated these groups separately because differences have been observed with other risk factors, such as body mass index, according to menopausal status (Cheraghi et al. 2012). These differences may be attributed to endogenous hormonal production and to other factors that are not well established. BC is a heterogeneous disease with potentially different etiologies in pre- and postmenopausal women; therefore, the evaluation of risk factors among these subgroups may have relevant public health implications.

The evaluation of BC’s association with nitrate and other environmental pollutants in different exposure periods is required because there is evidence suggesting that early exposure (e.g., before the first full-term pregnancy) might be the most relevant for inducing breast carcinogenesis (Brody et al. 2007). Although we evaluated three different exposure periods, we did not observe differences in the associations, most likely because of high correlations between exposure levels at different periods. In addition, we did not evaluate early-life exposure owing to a lack of data. This evaluation is warranted in future studies, particularly in settings with more available historical nitrate measurements in drinking water.

In the present study, dietary ingested nitrate levels were lower than levels observed in previous studies on this topic (Inoue-Choi et al. 2012), which may partly explain the lack of statistically significant associations. Our results suggested an inverse association between BC and ingested nitrate from vegetable sources. Vegetables contain endogenous nitrosation inhibitors (e.g., vitamins C and E), which may explain these results. Previous studies (Hord et al. 2009) have suggested beneficial health effects of nitrate from vegetable sources, which might also explain these results. Further research is needed to confirm these effects and to understand the underlying mechanisms.

Potential exposure misclassification is a concern in our study because most of the long-term nitrate levels in drinking water were imputed, particularly before 1980, and we did not account for water intake outside the home. However, the reported amount of water consumed at work (mean ± SD: 0.2 ± 0.3 L/day) and other places (0.01 ± 0.05 L/day) was smaller than that consumed at home (1.2 ± 0.7 L/day), and minor bias was expected. We conducted sensitivity analyses excluding women with the lowest interview quality, and slightly higher ORs were found, particularly among postmenopausal women. Changes in the type of water consumed, particularly in recent residences, may have led to exposure misclassification. To address this possibility, we calculated an alternative variable of waterborne ingested nitrate, which was described in “Methods.” In the analysis of this alternative variable, few women (*n* = 4 postmenopausal women and *n* = 6 premenopausal women) changed exposure categories, so the associations observed (see Table S2) were similar to the main results. Potential confounding by other environmental contaminants with estrogenic action and correlated to nitrate in drinking water may have occurred, although available data on selected pesticides (e.g., simazine, atrazine, terbuthylazine) showed levels below or around the quantification limit. Additionally, waterborne nitrite exposure was not evaluated because the available measurements showed unquantifiable or extremely low levels of nitrite.

Dietary nitrate estimations may also be prone to error because nitrate content was not available for some food items, and other relevant data, including vegetable storage and processing (i.e., washing, peeling and cooking), were not collected. Dietary nitrite intake was not evaluated; however, the lack of this information is not a major limitation because the main exposure route is through the endogenous reduction of nitrate (IARC 2010). Finally, because dietary information was collected with an FFQ, ingested nitrate misclassification because of recall bias may be a concern.

The matched case–control design by area of residence may lead to overmatching in environmental studies, which may have occurred in this study. However, overmatching would not affect the validity of our results (Agudo and González 1999). Controls had a higher education level than did the general population, which may hamper the external validity of the results. The heterogeneity of effects between some of the study areas may also be a limitation for the combined analyses.

A major strength of this study was the availability of detailed individual information on residential history, water consumption habits, and relevant covariables. Despite the limitations, the environmental data collected enabled us to evaluate BC associations for a long-term exposure window (from 18 years of age to 2 years before the study interview), in recent years, and in early adulthood. The information provided by the FFQ allowed us to evaluate nitrate ingestion from different dietary sources and to evaluate several potential confounders and effect modifiers that were not previously evaluated, including endogenous nitrosation modulators.

## Conclusion

Waterborne nitrate ingestion at the exposure levels observed was not associated with BC overall. However, BC was more common among postmenopausal women with the highest waterborne nitrate and red meat intake than among women with low waterborne nitrate and low red meat intake. Dietary nitrate was not associated with BC regardless of the exposure source or of menopausal status.

## Supplemental Material

(598 KB) PDF

## References

[r1] Agudo A, González CA (1999). Secondary matching: a method for selecting controls in case-control studies on environmental risk factors.. Int J Epidemiol.

[r2] Bingham SA, Hughes R, Cross AJ (2002). Effect of white versus red meat on endogenous N-nitrosation in the human colon and further evidence of a dose response.. J Nutr.

[r3] Brody JG Aschengrau A McKelvey W Swartz CH Kennedy T Rudel RA 2006 Breast cancer risk and drinking water contaminated by wastewater: a case control study. Environ Health 5 28, doi:10.1186/1476-069X-5-28 17026759 PMC1622744

[r4] Brody JG, Moysich KB, Humblet O, Attfield KR, Beehler GP, Rudel RA (2007). Environmental pollutants and breast cancer: epidemiologic studies.. Cancer.

[r5] Castaño-Vinyals G Aragonés N Pérez-Gómez B Martín V Llorca J Moreno V 2015 Population-based multicase-control study in common tumors in Spain (MCC-Spain): rationale and study design. Gac Sanit 29 4 308 315, doi:10.1016/j.gaceta.2014.12.003 25613680

[r6] Cheraghi Z Poorolajal J Hashem T Esmailnasab N Doosti Irani A 2012 Effect of body mass index on breast cancer during premenopausal and postmenopausal periods: a meta-analysis. PLoS One 7 12 e51446, doi:10.1371/journal.pone.0051446 23236502 PMC3517558

[r7] EFSA (European Food Safety Authority) (2008). Vegetables: Scientific Opinion of the Panel on Contaminants in the Food Chain (Question No. EFSA-Q-2006-071).. EFSA J.

[r8] Espejo-Herrera N, Kogevinas M, Castaño-Vinyals G, Aragonés N, Boldo E, Ardanaz E (2013). Nitrate and trace elements in municipal and bottled water in Spain.. Gac Sanit.

[r9] EU (European Union) (1998). Council Directive 98/83/EC on the Quality of Water Intended for Human Consumption.. OJ L.

[r10] Farran A, Zamora R, Cervera PC (2004). Tablas de composición de alimentos del d’Ensenyament Superior de Nutrició i Dietètica (CESNID) [in Spanish]..

[r11] Ferlay J, Steliarova-Foucher E, Lortet-Tieulent J, Rosso S, Coebergh J, Comber H (2013). Cancer incidence and mortality patterns in Europe: estimates for 40 countries in 2012.. Eur J Cancer.

[r12] Griesenbeck JS Steck MD Huber JJ Jr Sharkey JR Rene AA Brender JD 2009 Development of estimates of dietary nitrates, nitrites, and nitrosamines for use with the Short Willet Food Frequency Questionnaire. Nutr J 8 16, doi:10.1186/1475-2891-8-16 19348679 PMC2669451

[r13] Hankinson SE, Colditz GA, Willett WC (2004). Towards an integrated model for breast cancer etiology: the lifelong interplay of genes, lifestyle, and hormones.. Breast Cancer Res.

[r14] Hord NG, Tang Y, Bryan NS (2009). Food sources of nitrates and nitrites: the physiologic context for potential health benefits.. Am J Clin Nutr.

[r15] IARC (International Agency for Research on Cancer) (2010). Ingested nitrate and nitrite, and cyanobacterial peptide toxins.. IARC Monogr Eval Carcinog Risk Hum 94.

[r16] Inoue-Choi M, Ward MH, Cerhan JR, Weyer PJ, Anderson KE, Robien K (2012). Interaction of nitrate and folate on the risk of breast cancer among postmenopausal women.. Nutr Cancer.

[r17] Jakszyn P, Agudo A, Ibáñez R, García-Closas R, Pera G, Amiano P (2004). Development of a food database of nitrosamines, heterocyclic amines, and polycyclic aromatic hydrocarbons.. J Nutr.

[r18] Lijinsky W, Kovatch RM, Saavedra JE (1992). Carcinogenesis and mutagenesis by N-nitroso compounds having a basic center.. Cancer Lett.

[r19] Martin-Moreno JM, Boyle P, Gorgojo L, Maisonneuve P, Fernandez-Rodriguez JC, Salvini S (1993). Development and validation of a food frequency questionnaire in Spain.. Int J Epidemiol.

[r20] Pollán M, Pastor-Barriuso R, Ardanaz E, Argüelles M, Martos C, Galcerán J (2009). Recent changes in breast cancer incidence in Spain, 1980–2004.. J Natl Cancer Inst.

[r21] Romieu I Scoccianti C Chajès V de Batlle J Biessy C Dossus L 2015 Alcohol intake and breast cancer in the European Prospective investigation into Cancer and Nutrition. Int J Cancer 137 8 1921 1930, doi:10.1002/ijc.29469 25677034 PMC6300114

[r22] Stewart BW, Wild CP (editors) (2014). World Cancer Report 2014..

[r23] Tsubura A, Lai YC, Miki H, Sasaki T, Uehara N, Yuri T (2011). Review: animal models of *N*-Methyl-*N*-nitrosourea-induced mammary cancer and retinal degeneration with special emphasis on therapeutic trials.. In Vivo.

[r24] Veselik DJ, Divekar S, Dakshanamurthy S, Storchan GB, Turner JM, Graham KL (2008). Activation of estrogen receptor-α by the anion nitrite.. Cancer Res.

[r25] Wakida FT, Lerner DN (2005). Non-agricultural sources of groundwater nitrate: a review and case study.. Water Res.

[r26] Ward MH deKok TM Levallois P Brender J Gulis G Nolan BT 2005 Workgroup report: drinking-water nitrate and health—recent findings and research needs. Environ Health Perspect 113 11 1607 1614, doi:10.1289/ehp.8043 16263519 PMC1310926

[r27] Weyer PJ, Cerhan JR, Kross BC, Hallberg GR, Kantamneni J, Breuer G (2001). Municipal drinking water nitrate level and cancer risk in older women: the Iowa Women’s Health Study.. Epidemiology.

[r28] WHO (World Health Organization) (2008a). International Classification of Diseases (10th Revision) Online versions. Version 2008.. http://www.who.int/classifications/icd/icdonlineversions/en/.

[r29] WHO (2008b). Nitrate and nitrite.. In: Guidelines for Drinking Water Quality: Incorporating 1st and 2nd Addenda, Vol. 1, Recommendations. 3rd ed.

