# Supplementary material for: Ingested Nitrate and Breast Cancer in the Spanish Multicase-Control Study on Cancer (MCC-Spain)
Source: Environ Health Perspect. 2016 Mar 4;124(7):1042–9. doi: 10.1289/ehp.1510334 (PMC4937871; doi:10.1289/ehp.1510334)
Supplement: (598 KB) PDF [file ehp.1510334.s001.acco.pdf]

Environ Health Perspect
DOI: 10.1289/ehp.1510334

Note to readers with disabilities: *EHP* strives to ensure that all journal content is accessible to all readers. However, some figures and Supplemental Material published in *EHP* articles may not conform to 508 standards due to the complexity of the information being presented. If you need assistance accessing journal content, please contact ehp508@niehs.nih.gov. Our staff will work with you to assess and meet your accessibility needs within 3 working days.

Supplemental Material

Ingested Nitrate and Breast Cancer in the Spanish Multicase-Control Study on Cancer (MCC-Spain)

Nadia Espejo-Herrera, Esther Gracia-Lavedan, Marina Pollan, Nuria Aragonés, Elena Boldo, Beatriz Perez-Gomez, Jone M. Altzibar, Pilar Amiano, Ana Jiménez Zabala, Eva Ardanaz, Marcela Guevara, Antonio J. Molina, Juan Pablo Barrio, Ines Gómez-Acebo, Adonina Tardón, Rosana Peiró, Mª Dolores Chirlaque, Margarita Palau, Montse Muñoz, Laia Font-Ribera, Gemma Castaño-Vinyals, Manolis Kogevinas, and Cristina M. Villanueva

Table of Contents

Table S1. General characteristics of analyzed compared to excluded population

Table S2. Average waterborne ingested nitrate in different exposure periods and breast cancer association (OR and 95%CIs) by menopausal status

Table S3. Interaction of waterborne ingested nitrate with relevant covariables and breast cancer association (OR and 95%CI) among premenopausal women

Table S4. Waterborne ingested nitrate and breast cancer association (OR and 95%CI) stratified by other covariables among pre and postmenopausal women

Table S5. Breast cancer association (OR and 95%CI) with dietary ingested nitrate (mg/day) from different sources by menopausal status

Figure S1. Exposure-response relationship between waterborne nitrate intake (mg/day) from age 18 to 2 years before the interview and breast cancer association. Generalized additive models (GAMs) by study area

Table S1. General characteristics of analyzed (N=2765) compared to excluded (N=642) population

Characteristic		Excluded	Analyzed	*p value*
		n (%)	n (%)	
**Controls**		302 (47.0)	1,520 (55.0)	
**Cases**		340 (53.0)	1,245 (45.0)	<0.001
**Age**				
	Mean (SD)	55.8 (14.2)	58.4(12.7)	<0.001*
	Range	24-85	23-85	
**Education**				
	<Primary school	110 (17.1)	450 (16.3)	
	Primary school	176 (27.4)	899 (32.5)	
	Secondary school	206 (32.1)	871 (31.5)	
	University	150 (23.4)	545 (19.7)	0.046
**Body mass index**				
	<18.5	12 (1.9)	54 (2.0)	
	18.5-24.9	294 (45.8)	1,272 (46.0)	
	25-29.9	212 (33.0)	940 (34.0)	
	≥30	109 (17.0)	499 (18.0)	
	Missing	15 (2.3)	0 (0.00)	0.974
**Physical activity**				
	Low	476 (74.1)	1,958 (70.8)	
	Intermediate	65 (10.1)	356 (12.9)	
	High	101 (15.7)	451 (16.3)	0.128
**Family history of BC** [a]				
	No	458 (71.3)	2,005 (72.5)	
	Yes	154 (24.0)	664 (24.0)	
	Missing	30 (4.7)	96 (3.5)	0.883
**Age at menarche**				
	Mean (SD)	13(1.6)	12.8(1.6)	0.040*
	Range	8-18	7-20	
**Age at first birth** [b]				
	≤30 years	386 (78.6)	1768 (78.6)	
	>30 years	97 (19.8)	460 (20.5)	
	Missing	8 (1.6)	20 (0.9)	0.781
**Age at menopause** [c]				
	≤50 years	231 (56.6)	1,094 (52.6)	
	>50 years	121 (29.7)	685 (32.9)	
	Missing	56 (13.7)	303 (14.6)	0.144
**Menopausal status**				
	Premenopausal	232 (36.1)	679 (24.6)	

Postmenopausal	410 (63.9)	2086 (75.4)	<0.001
**Oral contraceptives use**			
Never	330 (51.4)	1,450 (52.4)	
Ever	306 (47.7)	1,311 (47.4)	
Missing	6 (0.9)	4 (0.1)	0.774
**Parity**			
Nulliparity	151 (23.52)	517 (18.70)	
1 delivery	118 (18.38)	468 (16.93)	
2 delivery	210 (32.71)	1130 (40.87)	
>2 deliveries	157 (24.45)	642 (23.22)	
Missing	6 (0.9)	8 (0.3)	0.001
**Energy intake**			
≤1479 kcal/day	145 (22.6)	735 (26.6)	
>1479-1894 kcal/day	177 (27.6)	809 (29.3)	
>1894 kcal/day	200 (31.2)	880 (31.8)	
Missing	120 (18.7)	341 (12.3)	0.487
**Red meat intake**			
<16 g/day	183 (28.5)	758 (27.4)	
>16-29 g/day	153 (23.8)	793 (28.7)	
>29 g/day	186 (29.0)	873 (31.6)	
Missing	120 (18.7)	341 (12.3)	0.174
**Processed meat**			
<5.2 g/day	177 (27.6)	740 (26.8)	
>5.2-13.4 g/day	173 (27.0)	807 (29.2)	
>13.4 g/day	172 (26.8)	877 (31.7)	
Missing	120 (18.7)	341 (12.3)	0.242
**Vitamin C intake**			
<129 mg/day	180 (28.0)	843 (30.5)	
129-203 mg/day	141 (22.0)	764 (27.6)	
>203 mg/day	201 (31.3)	817 (29.6)	
Missing	120 (18.7)	341 (12.3)	0.060
**Vitamin E intake**			
<8.6 mg/day	158 (24.6)	803 (29.0)	
>8.6-12.2 mg/day	150 (23.4)	801 (29.0)	
>12.2 mg/day	214 (33.3)	820 (29.7)	
Missing	120 (18.7)	341 (12.3)	0.007
**Waterborne ingested nitrate**			
<2.6 mg/day	146 (22.7)	930 (33.6)	
≥2.6-6.0 mg/day	167 (26.0)	908 (32.8)	
>6.0 mg/day	186 (29.0)	927 (33.5)	
Missing	143 (22.3)	0 (0.00)	0.120

[a] BC (Breast cancer). [b] Distribution among non-nulliparous women. [c] Distribution only for postmenopausal women. *p*-values for Chi square test in categorical variables and for T test* in continuous variables.

Table S2. Average waterborne ingested nitrate in different exposure periods and breast cancer association (OR and 95% CIs) by menopausal status.

Exposure periods	Ingested nitrate	Cases	Controls	Postmenopausal OR [b] (95%CI)	Ingested nitrate	Cases	Controls	Premenopausal OR [c] (95%CI)
**Long-term (age 18 years to 2 years before interview)[a]**								
	<2.2 mg/day	227	294	Ref.	<1.8 mg/day	71	87	Ref.
	≥2.2-3.8 mg/day	229	293	1.07 (0.83, 1.40)	≥1.8-3.1 mg/day	87	87	1.33 (0.84, 2.11)
	>3.8-9.1 mg/day	226	293	1.07 (0.81, 1.42)	>3.1-6.0 mg/day	80	87	1.02 (0.63, 1.65)
	>9.1 mg/day	231	293	1.31 (0.91, 1.91)	>6.0 mg/day	94	86	1.27 (0.73, 2.23)
	p for trend			*0.23*				*0.61*
**Recent (from 15 to 2 years before interview)**								
	<2.2 mg/day	218	290	Ref.	<2.0 mg/day	85	87	Ref.
	≥2.2-3.7 mg/day	226	292	1.08 (0.83, 1.40)	≥2.0-3.2 mg/day	75	86	0.97 (0.61, 1.52)
	>3.7-8.5 mg/day	227	288	1.18 (0.89, 1.57)	>3.2-6.2 mg/day	79	86	0.80 (0.50, 1.29)
	>8.5 mg/day	228	289	1.30 (0.93, 1.82)	>6.2 mg/day	90	86	1.01 (0.60, 1.71)
	p for trend			*0.11*				*0.81*
**Early adulthood (age 18 to 30 years)**								
	<2.2 mg/day	220	292	Ref.	<1.7 mg/day	78	87	Ref.
	≥2.2-4.2 mg/day	227	271	1.16 (0.89, 1.52)	≥1.7-3.0 mg/day	80	86	1.05 (0.66, 1.67)
	>4.2-9.2 mg/day	230	281	1.14 (0.86, 1.52)	>3.0-5.6 mg/day	71	86	0.83 (0.51, 1.34)
	>9.2 mg/day	209	281	1.12 (0.78, 1.61)	>5.6 mg/day	102	86	1.33 (0.78, 2.27)
	p for trend			*0.46*				*0.58*

[a] Analysis with alternative waterborne ingested levels, calculated assuming that women with bottled water consumption in recent residences, actually used tap water before the year 2000 and bottled water thereafter. [b] Adjusted for: study area, age, education, body mass index, family history of breast cancer, age at menopause, age at first birth, oral contraceptives use, and energy intake. [c] Age at menopause was excluded from the adjustment for premenopausal women. Trend *p*-values derived from a likelihood ratio test that comparing a model with the categorical nitrate intake variable as an ordinal variable (0, 1, 2) with a model that excluded this variable.

Table S3. Interaction of waterborne ingested nitrate with relevant covariables and breast cancer association (OR and 95%CI) among premenopausal women [a]

Waterborne Ingested nitrate	Cases	Controls	OR [b] (95% CI)	Cases	Controls	OR [b] (95% CI)	Interaction *p value**
	**Vitamin C**		**<142 mg/day**			**≥142 mg/day**	
<2.3 mg/day	54	55	Ref.	45	50	0.69 (0.38, 1.26)	
≥2.3-4.7 mg/day	43	52	0.88 (0.49, 1.61)	43	56	0.56 (0.31, 1.02)	
>4.7 mg/day	51	48	0.94 (0.49, 1.83)	51	48	0.84 (0.44, 1.61)	0.71
	**Vitamin E**		**<10 mg/day**			**≥10 mg/day**	
<2.3 mg/day	50	55	Ref.	49	50	0.77 (0.41, 1.46)	
≥2.3-4.7 mg/day	39	56	0.73 (0.40, 1.32)	47	52	0.71 (0.37, 1.38)	
>4.7 mg/day	44	44	1.10 (0.56, 2.15)	58	52	0.78 (0.39, 1.55)	0.75
	**Vitamin C+E**		**<152 mg/day**			**≥152 mg/day**	
<2.3 mg/day	54	56	Ref.	45	49	0.72 (0.39, 1.32)	
≥2.3-4.7 mg/day	43	51	0.96 (0.53, 1.74)	43	57	0.60 (0.33, 1.08)	
>4.7 mg/day	49	47	1.00 (0.52, 1.93)	53	49	0.94 (0.50, 1.78)	0.64
	**Folate**		**<276 µg/day**			**≥276 µg/day**	
<2.3 mg/day	47	59	Ref.	52	46	1.08 (0.58, 1.99)	
≥2.3-4.7 mg/day	38	53	0.94 (0.51, 1.72)	48	55	0.78 (0.43, 1.44)	
>4.7 mg/day	43	43	1.17 (0.59, 2.32)	59	53	1.02 (0.54, 1.95)	0.81
	**Red meat**		**<25 g/day**			**≥25 g/day**	
<2.3 mg/day	44	46	Ref.	54	59	0.97 (0.53, 1.75)	
≥2.3-4.7 mg/day	39	54	0.75 (0.40, 1.42)	46	54	0.85 (0.46, 1.59)	
>4.7 mg/day	46	55	0.85 (0.44, 1.66)	56	41	1.23 (0.61, 2.45)	0.64
	**Processed meat**		**<14 g/day**			**≥14 g/day**	
<2.3 mg/day	49	52	Ref.	50	53	0.82 (0.45, 1.49)	
≥2.3-4.7 mg/day	49	51	0.98 (0.54, 1.77)	37	57	0.57 (0.30, 1.06)	
>4.7 mg/day	39	52	1.28 (0.39, 1.41)	63	44	1.22 (0.62, 2.41)	0.05
	**Smoking**		**No**			**Yes**	
<2.3 mg/day	37	44	Ref.	74	72	1.37 (0.77, 2.45)	
≥2.3-4.7 mg/day	35	47	0.96 (0.50, 1.86)	63	68	1.19 (0.65, 2.17)	
>4.7 mg/day	52	45	1.29 (0.65, 2,56)	70	70	1.31 (0.69, 2,49)	0.74

[a] Only women with complete information on dietary covariables (N=596) and smoking (N=677) were analyzed. [b] Adjusted for: study area, age, education, body mass index, family history of breast cancer, age at first birth, and energy intake. **p*-value for overall interaction calculated by comparing the multivariate models with and without the interaction term using a likelihood ratio test

Table S4. Waterborne ingested nitrate and breast cancer association (OR and 95%CI) stratified by other covariables among pre and postmenopausal women

Waterborne ingested nitrate	Cases	Controls	OR [a] (95% CI)	Cases	Controls	OR [a] (95% CI)
**Histological type**			**Ductal [b]**			**Others/undefined [c]**
<2.6 mg/day	332	507	Ref.	91	507	Ref.
≥2.6-6.0 mg/day	289	507	0.93 (0.75, 1.16)	112	507	1.16 (0.84, 1.60)
>6.0 mg/day	330	506	1.16 (0.88, 1.52)	91	506	1.06 (0.70, 1.61)
**Estrogen receptor [d]**			**Negative**			**Positive**
<2.6 mg/day	73	507	Ref.	333	507	Ref.
≥2.6-6.0 mg/day	68	507	0.93 (0.64, 1.34)	321	507	1.02 (0.83, 1.26)
>6.0 mg/day	77	506	1.01 (0.63, 1.61)	336	506	1.16 (0.89, 1.52)

[a] Adjusted for: study area, age, education, body mass index, family history of breast cancer, age at first birth, use of oral contraceptives and energy intake. [b] ICD-10 code: C50. [c] ICD-10 codes: D05.1 and D05.7. [d] Numbers do not add 2765 due to missing data (N=37).

Table S5. Breast cancer association (OR and 95%CI) with dietary ingested nitrate (mg/day) from different sources by menopausal status

			Postmenopausal [a]				Premenopausal [a]
**Nitrate intake sources**	**Cases**	**Controls**	**OR (95%CI)**	**Nitrate intake**	**Cases**	**Controls**	**OR (95%CI)**
**Animal**							
<3.8 mg/day	228	344	Ref. [b]	<4.4 mg/day	73	103	Ref. [c]
3.8-5.8mg/day	266	344	0.98 (0.76, 1.27)	4.4-6.4mg/day	108	104	1.38 (0.89, 2.13)
> 5.8mg/day	303	343	1.03 (0.78, 1.35)	> 6.4mg/day	106	102	1.10 (0.68, 1.76)
**Vegetables**							
<80mg/day	284	344	Ref. [b]	<63mg/day	91	103	Ref. [c]
80-127 mg/day	269	344	0.91 (0.72, 1.16)	63-106 mg/day	93	104	0.88 (0.58, 1.35)
>127mg/day	244	343	0.81 (0.63, 1.05)	>106mg/day	103	102	0.89 (0.58, 1.37)
**Total diet**							
<94mg/day	281	344	Ref. [b]	<76 mg/day	92	103	Ref. [c]
94-143 mg/day	263	344	0.90 (0.71, 1.15)	76-118 mg/day	88	104	0.83 (0.54, 1.27)
>143mg/day	253	343	0.84 (0.64, 1.08)	>118 mg/day	107	102	0.91 (0.59, 1.40)
**Total diet+waterborne**							
<100mg/day	293	344	Ref. [b]	<81 mg/day	125	136	Ref. [c]
100-150 mg/day	255	344	0.81 (0.63, 1.03)	81-122 mg/day	86	100	0.94 (0.62, 1.44)
>150mg/day	249	343	0.79 (0.61, 1.03)	>122 mg/day	76	73	1.00 (0.65, 1.55)

[a] Only women with available data from the food frequency questionnaire were analyzed. [b] Adjusted for: study area, age, education, body mass index, family history of breast cancer, age at first birth, oral contraceptives use, age at menopause and energy intake. [c] Adjusted for the same covariates than OR for postmenopausal women, excluding age at menopause.

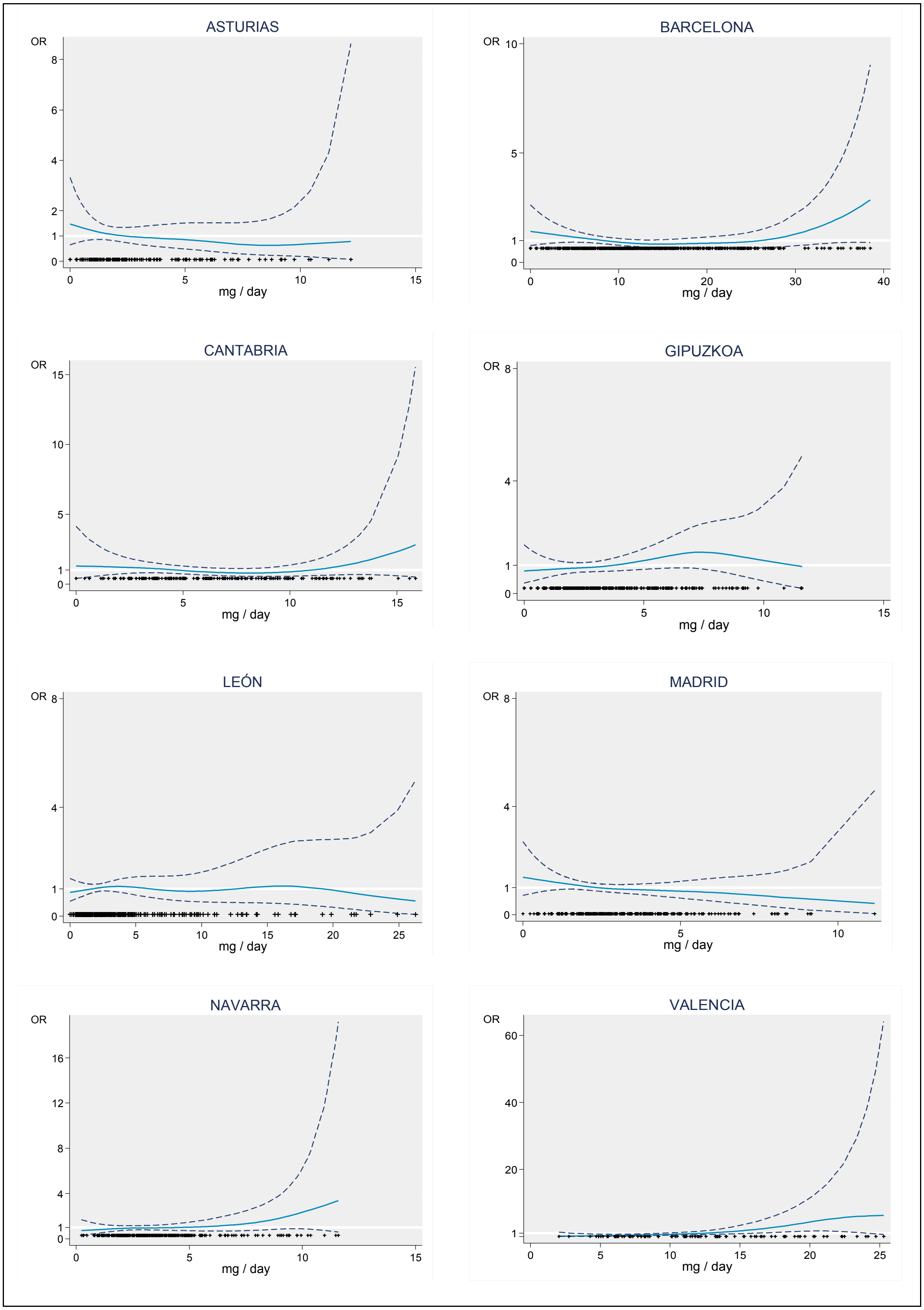

Figure S1. Exposure-response relationship between waterborne nitrate intake (mg/day) from age 18 to 2 years before interview and breast cancer. Generalized additive models (GAMs) by study area. Adjusted for: study area, age, education, body mass index, family history of breast cancer, age at first birth, use of oral contraceptives and energy intake.
